# Dietary energy and protein levels influenced the growth performance, ruminal morphology and fermentation and microbial diversity of lambs

**DOI:** 10.1038/s41598-019-53279-y

**Published:** 2019-11-12

**Authors:** Kai Cui, Minli Qi, Shiqin Wang, Qiyu Diao, Naifeng Zhang

**Affiliations:** Feed Research Institute, Chinese Academy of Agricultural Sciences, National Engineering Research Center of Biological Feed, Beijing, China

**Keywords:** Microbial ecology, Animal physiology

## Abstract

The aim of the study was to evaluate the ruminal function and microbial community of lamb under different nutrient levels. Sixty-four lambs with similarity body weight were randomly assigned to four groups after weaning off ewe’s milk on the 17th day (6.2 ± 0.2 kg). The lambs of the control group (CON) were fed a basal diet, and the other three groups were subjected to a diet of decreased protein (PR), digestible energy (ER) or both of them at 20% (BR) of basal diet. The decrease of dietary protein or energy level decreased the average daily gain, ruminal weight and mucosal thickness of lambs (*P* < 0.05). The total volatile fatty acid (TVFA), acetate and propionate concentration of the CON group were significantly higher than that of the other three groups. The dietary protein and energy level affected the microbial diversity, and low energy level increased the relative abundance of phyla of *Fibrobacteres*, whereas at the genus level, increased the relative abundance of *Butyrivibrio* and *Prevotellaceae*. Under different dietary energy and protein levels, 14 genera exhibited significant correlation with ruminal fermentation. These findings supplied new perspective for the understanding of the dietary effect on ruminal microbial community interactions and are of great significance for establishing the optimal nutrient supply strategy for lambs.

## Introduction

The rumen is a complex ecosystem that harbors a functional microbial population including bacteria, protozoa, archaea and fungi, which are important for the host’s nutrient uptake and energy metabolism^1^. The interaction between microorganisms and the host results in a symbiotic relationship that allows ruminants to digest diets rich in fiber^2^. Within this microbiome, bacteria are the dominant domain and make the greatest contribution to digestion and conversion of feed components to microbial proteins and volatile fatty acids (VFA), such as acetate, propionate, and butyrate during ruminal fermentation.

The microbiome is established as the development of rumen, and it is a widely held view in ruminant nutrition that rumen microbial populations highly adapt to different diets^3,4^. Studies in deer^5,6^, cows^7,8^, and lamb^9^ showed that interplay patterns between rumen bacterial community composition and metabolic phenotypes were altered by diets, and a large number of work have been performed to investigate how different dietary compositions and feeding programs can improve the ability of ruminal microbiota to degrade forages and feedstuffs for better animal performance and reduce the need for supplements. However, characteristic of ruminal fermentation and microbial community in response to different dietary nutritional levels are poorly understood in lamb.

In this study, we hypothesized that dietary with same ingredients but different nutritional levels could influence the development and function of rumen and microbial diversity of lamb. The aim of this research was to investigate the growth performance, ruminal fermentation and characterize the microbial composition and diversity of weaned lambs in response to dietary energy and protein levels based on high-throughput next generation sequencing. A better understanding of the correlation of nutritional level and ruminal development could provide the basis for the targeted improvement of nutrient levels in ruminants.

## Results

### Growth performance

No differences were detected in DM intake or nutrient intake of the milk replacer and starter among the four groups (*P* > 0.05; Table 1). The ADG and FCR of the lambs in BR group were significantly lower than that in the PR and ER group (*P* < 0.05), and there were no significant differences between PR and ER group (*P* > 0.05). The ADG and FCR of the three treatment group were significantly lower than the CON group (*P* < 0.01).

**Table 1:** Effects of nutritional levels on growth performance of early-weaned Hu lambs.

Items	Groups				SEM	*P*-value
	CON	PR	ER	BR		
Initial body weight	6.29	6.19	6.22	6.20	0.07	0.8241
Final body weight	15.40	13.90	13.94	13.02	0.24	0.0062
Average daily gain (g/d)	228.80^a^	186.56^b^	197.72^b^	168.55^c^	6.19	<0.0000
Milk replacer intake (g/d)	154.63	154.63	154.63	154.63	-	-
Starter intake (g/d)	325.96	322.26	337.22	320.26	3.36	0.3148
Feed conversion ratio	2.10^a^	2.57^b^	2.49^b^	2.82^c^	0.07	0.0002

^a,b,c^Means within same row with the same superscript letter are not significantly different (*P* > 0.05).

### Ruminal morphology and fermentation

The effects of energy and protein level on ruminal development and fermentation are shown in Table 2. The rumen weight of lambs in the CON group was significantly higher than the other three groups (*P < *0.05). The decrease of protein or/and energy had no effect on the length and width of papillae of the rumen (*P* > 0.05). The mucosal thickness of rumen of group PR, ER, and BR was significantly lower than that of the CON group (*P* < 0.05).

**Table 2:** Effects of nutritional levels on ruminal fermentation of early-weaned Hu lambs.

Items	Groups				SEM	*P*-value
	CON	PR	ER	BR		
**Rumen morphometrics**						
Rumen weight (g)	327.9^a^	285.73^b^	241.48^c^	237.43^c^	11.49	0.0003
Papillae Length (μm)	3247.34	3107.44	2741.46	2945.79	129.70	0.595
Papillae Width (μm)	394.28	383.85	341.63	322.21	14.00	0.2195
Mucosal thickness (μm)	211.31^a^	175.18^b^	140.89^b^	156.33^b^	8.53	0.0059
**Rumen fermentation**						
PH	6.78	6.89	6.96	6.95	0.16	0.8540
NH_3_^-^N (mmol/L)	17.47	15.60	13.42	12.39	3.60	0.8718
TVFA (mmol/L)	36.25^a^	10.14^b^	13.26^b^	14.16^b^	4.31	0.0055
Acetate (mmol/L)	18.23^a^	5.84^b^	5.27^b^	7.78^b^	1.70	0.0013
Propionate (mmol/L)	17.53^a^	3.06^b^	3.42^b^	4.23^b^	1.34	0.0001
Butyrate (mmol/L)	1.68^a^	0.58^b^	0.86^b^	1.01^ab^	0.18	0.0512

^a,b,c^Means within same row with the same superscript letter are not significantly different (*P* > 0.05).

Ruminal pH and NH_3_-N concentrations showed no significant difference among the four groups (*P* > 0.05). The TVFA, acetate and propionate concentration of the CON group was significantly higher than that of the other three groups (*P* < 0.05). Compared to the CON group, butyrate concentration of PR, ER and BR group showed a decreased tendency (*P* = 0.0512). No significant difference was observed among the PR, ER, and BR group (*P* > 0.05).

### Index of microbial community

**Figure 1:**
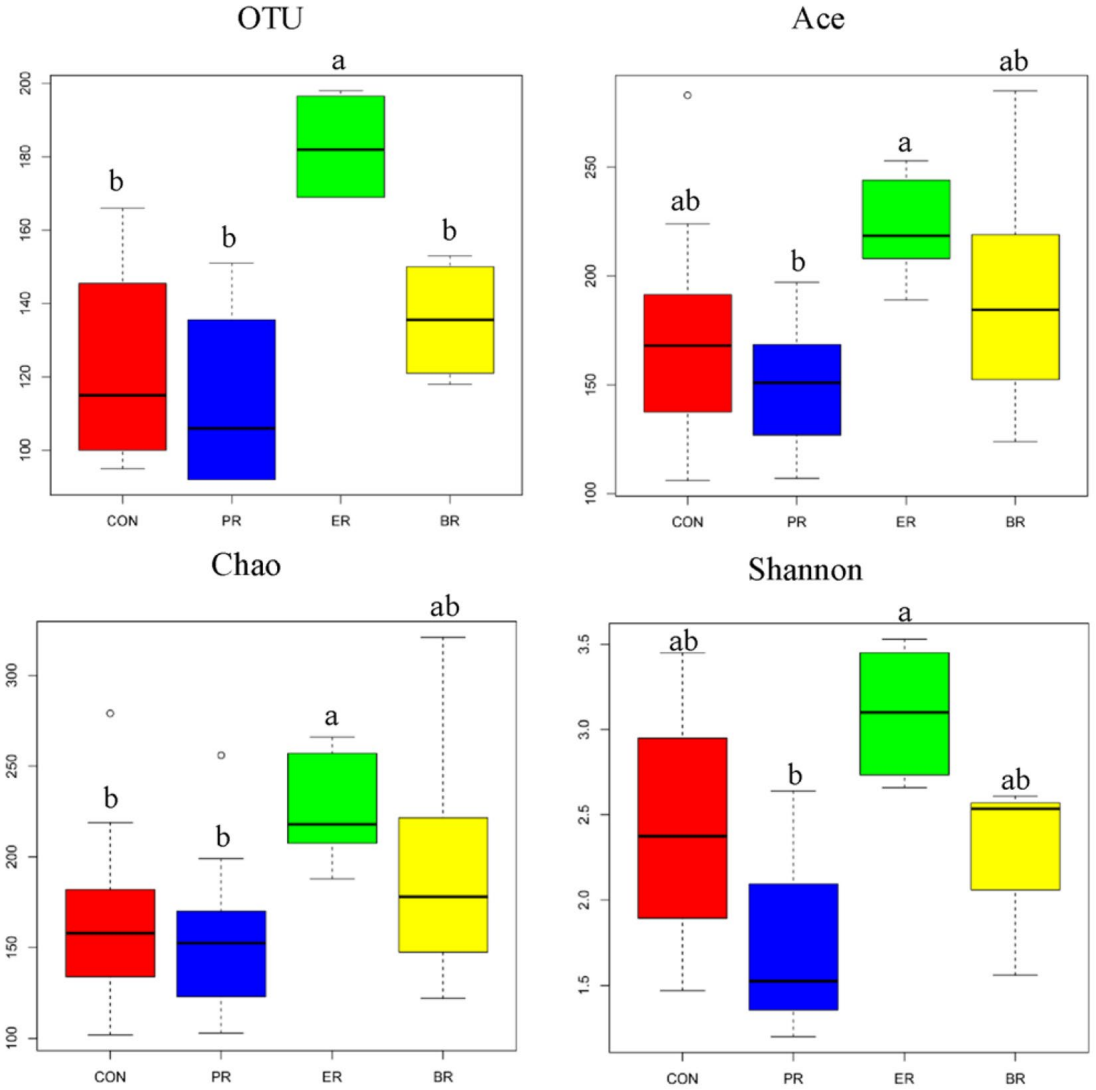
Community richness estimates and diversity indices for different treatments.

The saturation plateau of rarefaction curves indicated that the sampling effort had sufficient sequence coverage to accurately describe the bacterial composition of each group (Fig. S1). Based on OTU, indices of bacterial richness were estimated by the index of Ace and Chao, and indices of bacterial diversity were determined using the index of Simpson and Shannon. Dietary energy levels significantly altered the rumen bacterial community (Fig. 1). At the 0.03 dissimilarity level, the OTU numbers of ER group was significantly higher than the other three groups (*P* < 0.05). The indices of Chao and Shannon were significantly affected by the protein and energy levels (*P* < 0.05).

### The relative abundance and diversity of bacterial communities

**Figure 2:**
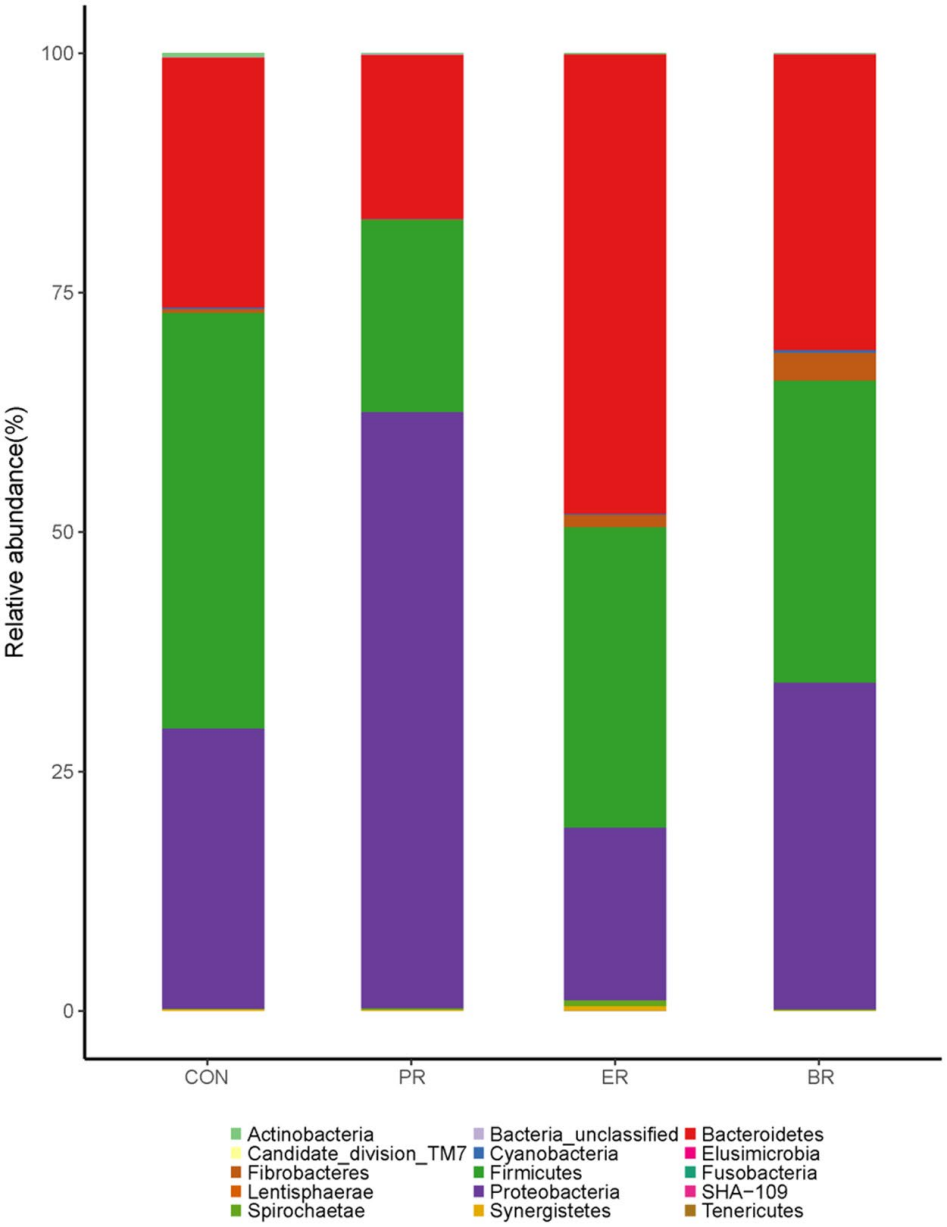
Phylum-level composition of the rumen microbiome. A color-coded bar plot showing the average bacterial phylum distribution across the different age groups that were sampled.

All sequences were classified from phylum to species based on the SILVA taxonomic database. Fifteen different phyla were detected in these samples. The four groups showed dissimilar taxonomic compositions at the phylum-level distributions and the major sequences obtained from the samples belonged to *Firmicutes, Bacteroidetes*, and *Proteobacteria* (Fig. 2, Table 3). The relative abundance of these predominant phyla varied considerably among the four groups. Compared with the CON and PR group, the phylum *Bacteroidetes* and *Fibrobacteres* were abundant in the samples taken from the ER and BR groups while the phylum *Fibrobacteres* of BR group was significantly higher than that of the other three groups (*P* < 0.05). The abundance of phylum *Firmicutes* and *Proteobacteria* showed no significant difference among the four groups while the abundance of phylum *Proteobacteria* showed an increased tendency (*P* = 0.0973). The other 9 phyla were relatively minor (<1% of total sequences) in abundance in comparison.

**Table 3:** Comparison of the dominant phylum (average relative abundance ≥1% for at least one group) within the rumen.

Items	Groups				SEM	*P*-value
	CON	PR	ER	BR		
*Bacteroidetes*	26.10	17.09	38.54	30.92	6.27	0.2038
*Fibrobacteres*	0.43^b^	0.12^b^	1.25^b^	3.90^a^	0.72	0.0325
*Firmicutes*	43.37	20.05	24.48	20.44	7.11	0.1374
*Proteobacteria*	29.26	62.26	18.04	34.15	11.45	0.0973

^a,b^Values in the same row with different superscripts differ significantly (*P* < 0.05).

**Figure 3:**
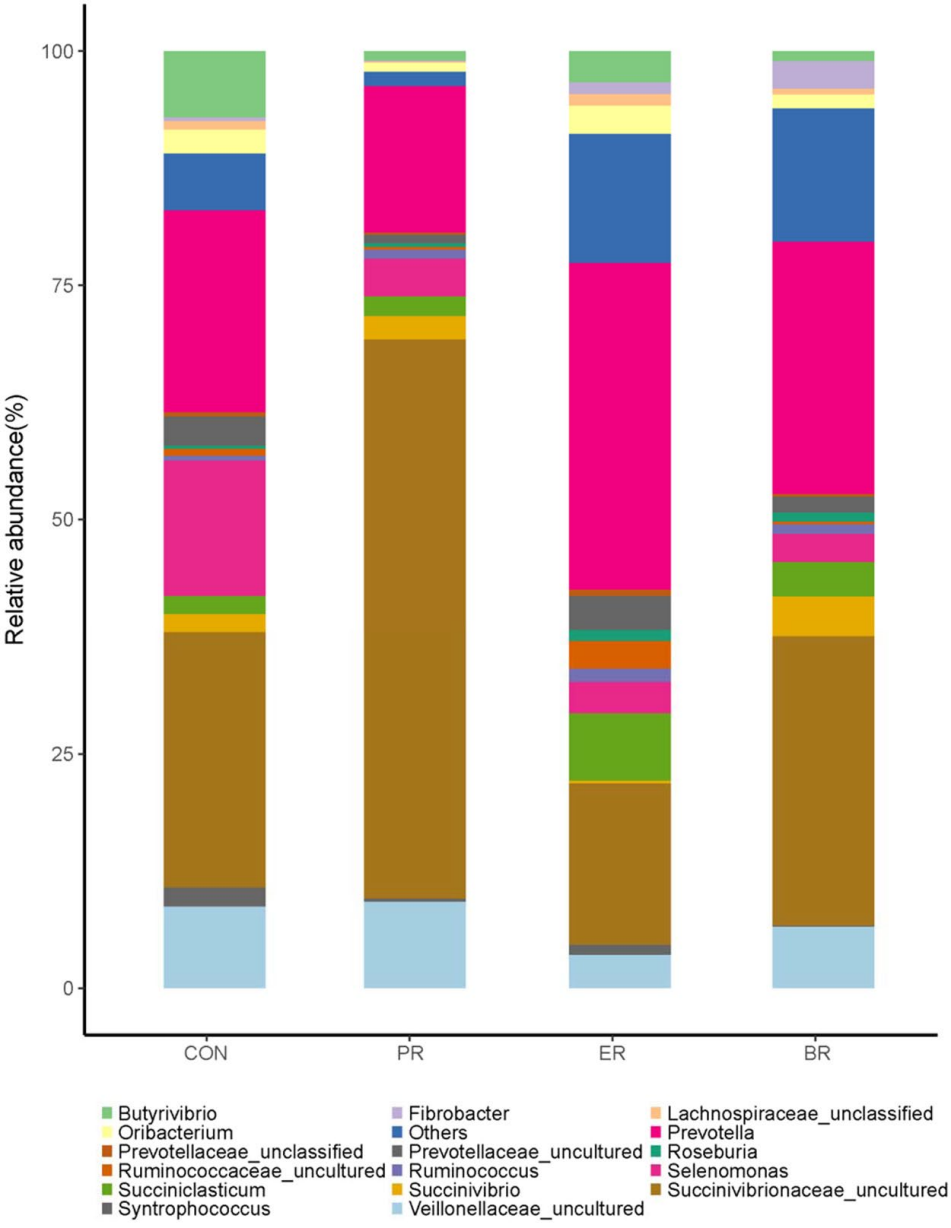
Phylum-level composition of the rumen microbiome. A color-coded bar plot showing the average bacterial phylum distribution across the different age groups that were sampled.

**Figure 4:**
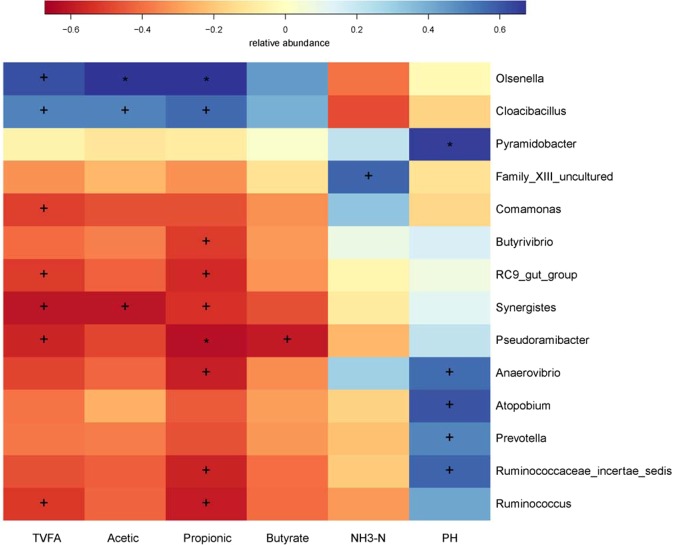
Spearman correlation analysis of VFA and microbiome at genus level. The depth of the color indicates the correlation between species and environmental factors. The “+” and “*” indicates the different level at 0.05 and 0.01, respectively.

At the genus level, 103 genera were detected in the samples. The most abundant genera (with a relative abundance ≥2% of the four libraries) were used to determine which bacteria might be the most important (Fig. 3, Table 4). The most abundant taxa of the CON group included *Prevotella, Selenomonas, Succiniclasticum*, as well as the unclassified taxa derived from *Succinivibrionaceae* (family) and *Veillonellaceae* (family). In the PR group, the *Succinivibrionaceae_uncultured* was predominant with an abundance of 59.62%, followed by *Prevotella*, *Veillonellaceae_uncultured*, *Selenomonas, Succiniclasticum* and *Succinivibrio*. In the ER group, the most dominant genera were *Prevotella, Succinivibrionaceae_uncultured, Succiniclasticum, Veillonellaceae_uncultured, Prevotellaceae_uncultured* and *Butyrivibrio*, which together accounted for 69.89% of the total sequences. The abundance of genus *Butyrivibrio* was abundant in the samples taken from the ER group when compared with the CON and PR groups. The samples of both protein and digestible energy reduced group showed that most of the dominant taxa were *Succinivibrionaceae_uncultured*, *Prevotella*, *Veillonellaceae_uncultured*, *Selenomonas, Succinivibrio*, and *Fibrobacter*. We evaluated the correlation between the ruminal fermentation parameters and bacteria at genus level by performing Spearman correlation analysis. Almost 14 genera were exhibited significant correlation with TVFA, acetic, butyrate, propionic, NH_3_-N and pH, respectively (Fig. 4).

**Table 4:** Comparison of the dominant genus (average relative abundance ≥2% for at least one group) within the rumen.

Taxa	Groups				SEM	*P*-value
	CON	PR	ER	BR		
*Succinivibrionaceae_uncultured*	35.44	59.62	22.96	41.16	11.15	0.2473
*Prevotella*	25.18	15.66	34.84	26.95	6.44	0.2703
*Veillonellaceae_uncultured*	11.56	9.25	4.75	6.58	3.86	0.7089
*Selenomonas*	14.43	4.03	3.34	3.02	0.61	0.2723
*Succiniclasticum*	1.96	2.07	7.19	3.67	1.61	0.1294
*Butyrivibrio*	0.29^b^	0.34^b^	1.43^a^	1.06^ab^	0.21	0.0187
*Prevotellaceae_uncultured*	3.12^a^	0.92^b^	3.68^a^	1.72^ab^	0.65	0.0404
*Succinivibrio*	1.91	2.53	0.27	4.21	1.59	0.4658
*Oribacterium*	2.59	0.97	3.01	1.44	1.13	0.6016
*Fibrobacter*	0.43	0.12	1.25	2.93	0.86	0.1798
*Ruminococcaceae_uncultured*	0.72	0.27	2.95	0.28	1.11	0.3083
*Syntrophococcus*	2.03	0.36	1.05	0.14	0.88	0.4530

^a,b^Values in the same row with different superscripts differ significantly (*P* < 0.05).

## Discussion

In this study, we evaluated the growth performance, ruminal development and fermentation, and microbial diversity of weaned lambs under different dietary energy and protein levels. Hossain *et al*. (2003) and Negesse *et al*. (2001) reported that energy and protein level of diets influenced the growth performance and N retention of goats, respectively^9–11^. Results from this study indicated that a lower level of protein, energy, or both protein and energy significantly decreased the average daily gain and feed efficiency. This result was consisted with the fact that high protein and energy intake was necessary to meet the demand of rapid growth of lamb. Atti *et al*. (2004) reported that lambs supplemented with high protein level diet exhibited higher growth rates than those fed diets of low protein level during the first 6 weeks^12^. Previous study indicated that high concentrate level (600 g) of diet improved the ADG and FCR of lambs^13^.

The establishment of rumen function is the symbol of the transition from functioning as a monogastric to a ruminant^14^. Rumen is the dominant place where bacteria contributed to digest and convert plant materials to volatile fatty acids and microbial proteins. The development of rumen is critical for the utilization of solid feed. In this study, low protein and energy diet significantly decreased the rumen weight and mucosal thickness. This finding is in consistent with the fact that young animals may require high protein and energy intakes to ensure the organ development. These observations are consistent with analogous trials on digestive tract development. In ruminants, gastrointestinal tissues are affected by changes in ME intake^14^, protein intake, as well as dietary energy density^15,16^. Most studies report height and width of ruminal papillae as an estimate of ruminal epithelium growth^17^. Length, uniformity, and general appearance of papillae are greatly influenced by composition and particle size of the diet. Previous studies showed that encouraging solid feed intake by restricting milk access increases the size of the rumen and the morphological structure of rumen papillae^18^. Shen *et al*. (2004) reported that an energy-rich diet caused ruminal papillae proliferation in young goats^19^. Wan *et al*. (2012) proved that the high concentrate level significantly increased both ruminal papillae length and width, compared with the hay and low concentrate feeding group^20^. In our research, decrease of protein or energy decreased the length and width of papillae, but no obvious difference was observed among the groups. The insufficiently limitation of protein or energy at 20% of basal diet might account for this fact. Previous studies have confirmed the VFA concentration tended to increase with increasing dietary energy level by adding more non-structure carbohydrates (NSC)^21^. Compared to the control group, low energy or protein level decreased the concentration of volatile fatty acid (VFA). These results are consistent with other researches^22^.

Ruminal microorganisms and host have evolved together for millions of years. The primary function of ruminal microbiome is the conversion of fibre into digestible compounds for the utilization of the ruminants. In the present study, 16S rRNA sequencing method was selected to evaluate the diversity of the ruminal bacterial community of lambs under different dietary energy and protein levels. The results of the present study revealed that *Bacteroidetes, Firmicutes* and *Proteobacteria* were the dominant phyla among the groups. Our findings were in agreement with previous studies that *Bacteroidetes* and *Firmicutes* are numerically the most dominant phyla in the microbiome of terrestrial mammals^23–26^.

The current research found that the genera *Succinivibrionaceae_uncultured, Prevotella, Veillonellaceae_uncultured, Selenomonas, Succiniclasticum*, and *Butyrivibrio* dominated in the four groups. *Prevotella* has been reported as the most abundant genus in the rumen of adult cows, which is related to ruminal carbohydrate and protein fermentation^27^. This research showed that the proportion of *Prevotellaceae_uncultured* in the PR group was significantly lower than that in the CON and ER group. Mao *et al*. (2012) indicated the abundance of *Prevotella* was highly correlated with the content of CP^7^. Since the content of CP in the alfalfa hay is greater than in the rice straw, this provided a reasonable explanation for the higher abundance of *Prevotella* observed in the alfalfa samples^28^. In this study, the *Butyrivibrio* abundance of ER and BR group was higher than the CON and PR group. The *Butyrivibrio* species which involved in a number of ruminal functions are common in the ruminants, such as deer, cows and sheep^29^. Of particular importance to ruminant digestion, the degradation of structural carbohydrates of plant materials is the most important role of *Butyrivibrio*. The dietary crude fiber content of ER and BR group was more than twice when compared to the CON and PR group, and the increased fiber level might be the reason for the high abundance of *Butyrivibrio*.

In conclusion, the results presented here provide new information regarding the effects of different dietary energy and protein levels on growth performance and ruminal development and microbiota communities. Low level of protein or energy retarded the growth performance and ruminal development of lambs. Low dietary energy levels significantly decreased the concentration of volatile fatty acids. Based on 16S rRNA gene sequencing method, this study indicated the changes of the overall composition of the bacterial communities in the rumen ecosystem. The dietary protein and energy level affected the microbial diversity and the low level of energy increased the relative abundance of *Fibrobacteres* at the phylum level and increased the relative abundance of *Butyrivibrio* and *Prevotellaceae* at the genus level, and 14 genera exhibited significant correlation with ruminal fermentation. These findings are of great importance for the targeted improvement of nutrient levels in ruminants.

## Materials and Methods

### Statement

This research was conducted at the Hai Lun sheep industry Co., Ltd., Jiangsu, China (latitude 32.30′N, longitude 119.54′E). All experiments were performed in accordance with relevant guidelines and regulations. The experimental protocol and all methods were approved by the Animal Ethics Committee of Chinese Academy of Agricultural Sciences, and humane animal care and handling procedures were performed throughout the experiment (AEC-CAAS-FRI-CAAS20180602).

### Animals, diets and management

Sixty-four lambs with an average birth weight of 2.5 ± 0.2 kg were randomly divided into four groups (n = 16/group) after weaning off ewe’s milk on the 17th day (6.2 ± 0.2 kg), with 4 replicates of each group and 4 lambs per replicate. The control group (CON) was fed a basal diet and the other three groups were subjected to a diet of decreased protein (PR), digestible energy (ER) or both protein and digestible energy at 20% (BR) of basal diet. Each group was fed the assigned milk replacer and starter from 21 to 60 days after 4 days transitional period of milk replacer and pelleted starter (Diameter, 4 mm; Length, 10 mm). These lambs were fed 3 times daily (08:00, 12:00, and 18:00 h) from day 21 to 30 and then twice daily (09:00, 18:00 h) from day 31 to 60, and the feed amount of milk replacer was adjusted in direct accordance with 2% of the lamb’s body weight. Milk replacer consumption was equal in all four groups. The pelleted starter supplement of the CON group was assigned to be fed ad libitum, and the feed amounts of the other three groups were according to the intake of the CON group. The feed intake of pelleted starter was kept consistent among the four groups. The components and chemical composition of the milk replacer and pelleted starter are presented in Table 5.

**Table 5:** Composition and nutrient levels of milk replacer and starters (DM basis).

Items	Milk replacer				Starter			
	CON	PR	ER	BR	CON	PR	ER	BR
**Ingredients**								
Corn	—	—	—	—	53	62	25	38
Soybean meal	—	—	—	—	27	14	27	16
Powdered rice hulls	—	—	—	—	0	0	16	17
Wheat bran	—	—	—	—	6	10	18	15
Premix^a^	—	—	—	—	4	4	4	4
Alfalfa meal	—	—	—	—	10	10	10	10
Total	—	—	—	—	100	100	100	100
**Nutrient levels** ^**b**^								
DM	94.35	94.51	93.33	93.48	86.59	86.50	87.35	87.25
CP	24.21	19.13	24.45	19.26	20.80	16.35	20.68	16.10
ME,MJ/Kg	14.83	14.77	12.55	12.55	10.59	10.61	8.52	8.52
EE	20.58	20.78	11.06	10.92	2.89	3.12	2.67	2.83
Ash	4.99	4.84	4.81	4.88	9.71	9.82	9.85	9.82
NDF	—	—	—	—	18.49	18.18	28.89	28.28
Calcium	0.95	0.95	0.95	0.95	0.41	0.40	0.51	0.46
Phosphorus	0.68	0.68	0.68	0.68	0.24	0.21	0.26	0.22

^a^The premix provided the following nutrients per kilogram of the diet: VA 12000IU, VD 20 00IU, VE 30IU, Cu 12 mg, Fe 64 mg, Mn 56 mg, Zn 60 mg, I 1.2 mg, Se 0.4 mg, Co 0.4 mg, Ca 3.2 g, P 1.2 g, NaCl 6.4 g.

^b^Nutrient levels are all measured values except ME. ME of milk replacer and starter was calculated according to Tables of Feed Composition and Nutritive Values in China 2012 and Feeding Standard of sheep(NY/T 816-2004); DM, dry matter; CP, crude protein; ME, metabolic energy; EE, ether extract; NDF, neutral detergent fiber.

### Chemical analyses

The lambs were weighed at 20, 40, and 60 days, and the intake of milk replacer and starter feed was measured daily to calculate the average daily gain (ADG), average daily feed intake (ADFI), and feed conversion ratio (FCR).

The composition and nutrient levels of milk replacer and starters were analyzed according to the official methods of analysis (Association of Official Analytical Chemists: Washington, DC)^30^. The dry matter was determined by drying the samples in an oven at 105 °C for 24 h (method 930.15; AOAC1990). The nitrogen (N) content was determined by the Kjeldahl method and crude protein (CP) was calculated as 6.25 × N (method 984.13; AOAC1990). The ether extract was measured by the weight loss of the dry matter upon extraction with diethyl ether in a Soxhlet extraction apparatus for 8 h (method 920.85; AOAC 1990). The contents of ash (550 °C in a muffle furnace for 6 h, method 942.05; AOAC1990), neutral detergent fiber (NDF; method 962.09; AOAC 1990) and gross energy (GE; Bomb calorimeter, C200; IKA Works Inc., Staufen, Germany) were determined using appropriate protocols. The calcium (Ca) was analysed using an atomic absorption spectrophotometer (M9 W-700; Perkin-Elmer Corp., Norwalk, CT, USA; method 968.08; AOAC1990). The phosphorus (P) was analysed by the molybdovanadatecolourimetric method (method 965.17; AOAC1990) using a spectrophotometer (UV-6100; Mapada Instruments Co., Ltd, Shanghai, China).

### Ruminal morphometrics

Four lambs (healthy and bodyweight close to the average bodyweight of the group) from each group were selected at the age of 60 days and slaughtered to collect the ruminal tissues and digesta. A 2-cm^2^ fragment of each rumen was collected from ventral sac and fixed in 4% paraformaldehyde (Sigma-Aldrich) for histological assessment. The samples were dehydrated with an ethanol and toluene (Beijing Chemical Works) series and embedded in paraffin (Leica, Wetzlar, Germany). Serial sections (6 μm thickness) were mounted on gelatin-coated glass slides and stained with hematoxylin and eosin (H&E)^31^. For histomorphometry, five ruminal papillae from each section were examined and measured (in µm) considering their papillae height, papillae width, and mucosal thickness. Both measurements were taken at 200x utilizing an Olympus BX51 photomicroscope, equipped with a DP software for the image analysis (Olympus, Italy).

### Ruminal fermentation parameters

Samples of ruminal digesta were collected at the age of 60 days after slaughtered. The pH of the ruminal fluid was determined using a pH metre (Model 144 PB-10, Sartorius Co., Germany). Approximately 10-ml sample of the strained fluid was collected and stored frozen at −20 °C for analysis of VFA and NH_3_-N after acidified with 2 ml 25% (w/v) metaphosphoric acid. The concentration of VFA in ruminal fluid was measured by gas chromatography (GC) using methyl valerate as an internal standard in an Agilent 6890 series GC equipped with a capillary column (30 m x0.53 mm internal diameter, film thickness 1/-Lm)^32^. Ammonia-N was measured with the method of the colorimetric method described by Ma *et al*. (2015)^33^.

### DNA extraction, amplification of 16S rRNA and Hiseq sequencing

Microbial DNA was extracted after ruminal samples were thoroughly homogenized with a commercial DNA extraction Kit (Omega Bio-tek, Norcross, GA, U.S.) according to manufacturer’s protocols. Amplification and sequencing were performed as described by Mao *et al*. (2012)^7^. The V3-V4 region of the bacteria 16S ribosomal RNA genes were amplified using primers 338F (5’-barcode-ACTCCTRCGGGAGGCAGCAG)-3’ and 806R (5’-GGACTACCVGGGTATCTAAT-3’), where the barcode is an eight-base sequence unique to each sample.

Amplicons were purified using the AxyPrep DNA Gel Extraction Kit (Axygen Biosciences, Union City, CA, U.S.) according to the manufacturer’s instructions and quantified using QuantiFluor™ -ST (Promega, U.S.). Purified amplicons were pooled in equimolar and paired-end sequenced (2 × 250) on an Illumina HiSeq platform (Illumina Inc., San Diego, CA) according to the standard protocols.

### Statistical and bioinformatics analysis

As described by Caporaso *et al*. (2010), the raw Illumina sequences data were demultiplexed, quality filtered, and analyzed using the Quantitative Insights into Microbial Ecology (QIIME, v.1.8.0)^34^. The assembled sequences were assigned to operational taxonomic units (OTU) at a 97% identity level using UPARSE after quality control^35^. The phylogenetic affiliation of 16S rRNA gene sequence was analyzed against the SILVA (SSU115) 16S rRNA database using Ribosomal Database Project (RDP) Classifier (http://rdp.cme.msu.edu/) with a confidence threshold of 70%^36,37^. Rarefaction curves, α diversity, and β diversity calculations were also performed using QIIME^38^. Spearman correlation analysis between bacterial and ruminal fermentation parameters was performed using R corrplot^39^. The sequencing data of this research was submitted to the Sequence Read Archive (SRA) with an accession number of SRP145573.

Differences in growth performance and ruminal morphology, fermentation parameters, and microbiota diversity among the four groups were analyzed using the SAS (version 9.1, SAS Institute, Inc., Cary, NC, USA; 2004) general linear model (GLM). The following model was fitted to the data: Y_i_ = μ + α_i_ + e_i_, where Y_i_ is the dependent variable; μ represents the overall mean; α_i_ represents the fixed effect of treatment, and e_i_ is the random residual error due to the replicate. Duncan’s Multiple Range Test was used to the statistical differences analysis among the means of the treatments. Treatment differences with *P* < 0.05 were considered statistically significant while 0.05 ≤ *P* < 0.10 was defined as a tendency.
